# Integration of Genetic and Phenotypic Data in 48 Lineages of Philippine Birds Shows Heterogeneous Divergence Processes and Numerous Cryptic Species

**DOI:** 10.1371/journal.pone.0159325

**Published:** 2016-07-21

**Authors:** Kyle K. Campbell, Thomas Braile, Kevin Winker

**Affiliations:** University of Alaska Museum and Department of Biology and Wildlife, Fairbanks, Alaska, United States of America; Instituto de Higiene e Medicina Tropical, PORTUGAL

## Abstract

The Philippine Islands are one of the most biologically diverse archipelagoes in the world. Current taxonomy, however, may underestimate levels of avian diversity and endemism in these islands. Although species limits can be difficult to determine among allopatric populations, quantitative methods for comparing phenotypic and genotypic data can provide useful metrics of divergence among populations and identify those that merit consideration for elevation to full species status. Using a conceptual approach that integrates genetic and phenotypic data, we compared populations among 48 species, estimating genetic divergence (*p*-distance) using the mtDNA marker ND2 and comparing plumage and morphometrics of museum study skins. Using conservative speciation thresholds, pairwise comparisons of genetic and phenotypic divergence suggested possible species-level divergences in more than half of the species studied (25 out of 48). In speciation process space, divergence routes were heterogeneous among taxa. Nearly all populations that surpassed high genotypic divergence thresholds were Passeriformes, and non-Passeriformes populations surpassed high phenotypic divergence thresholds more commonly than expected by chance. Overall, there was an apparent logarithmic increase in phenotypic divergence with respect to genetic divergence, suggesting the possibility that divergence among these lineages may initially be driven by divergent selection in this allopatric system. Also, genetic endemism was high among sampled islands. Higher taxonomy affected divergence in genotype and phenotype. Although broader lineage, genetic, phenotypic, and numeric sampling is needed to further explore heterogeneity among divergence processes and to accurately assess species-level diversity in these taxa, our results support the need for substantial taxonomic revisions among Philippine birds. The conservation implications are profound.

## Introduction

Despite difficulties of species delineation, species-level diversity is arguably the most important measure of biodiversity [1, 2]. Most of the world’s terrestrial vertebrate diversity has been described to the species level, and species recognition is important in conservation efforts and public awareness [3–5]. In the tropics, where species richness is highest and where much undiscovered biological diversity is believed to exist [6–8], cryptic diversity can be overlooked or obscured by taxonomy that often relies heavily on phenotypic characters [9–11]. Conventional species delimitation has relied on divergence exhibited in sympatry, wherein intrinsic barriers to gene flow provide evidence of species limits [12]. Species status for populations diverging in allopatry, such as island taxa, can be more difficult to ascertain [1, 13]. In many cases, differences between populations are not deemed sufficient for species-level recognition, causing these populations to be recognized as subspecies [14]. In island systems, individual islands often host endemic subspecies of wide-ranging species [3, 15]. Taxonomy of island populations is important as they are more prone to extinction than mainland populations; in birds the majority of recent extinctions have been of island endemic species [16, 17]. Island endemism is important for conservation planning; these efforts often focus on island endemics in tropical island systems, as in the Philippines [18, 19]. Conservation of island populations can be affected by inaccurate species delineations [10], making this an important conservation issue.

Divergence and speciation represent an inherently multidimensional process. Taxonomy based solely on phenotypic differences, or solely on stochastic genetic variation, reflects only one dimension of divergence between populations [20]. Taxonomy also uses bins to describe a continuous process. To aid us in accurately determining diversity around the taxonomic “bin” of species, we adopt an approach that integrates phenotypic and genotypic evidence. Through this integration, we can assess the need for taxonomic revision and also visualize the processes of divergence to understand how taxa might diverge in different ways towards speciation, e.g., remaining phenotypically cryptic despite deep genetic divergence, or, conversely, being dramatically different in phenotype despite shallow genetic divergence [9, 20]. We compare taxa in a divergence process space in which multiple general routes to speciation exist. In this space, populations diverging equivalently along both genetic and phenotypic axes represent one general route to speciation, and populations diverging more along one axis than the other represent two other general routes, one with rapid phenotypic divergence and the third with low phenotypic divergence relative to deeper genetic divergence [20].

Standardized scoring of phenotypic characters can identify divergent populations that may merit species-level elevation [13], and this approach has supported elevation of populations to full biological species status on islands, including the Philippines [21, 22]. This approach leverages data from comparisons of well-defined species pairs in sympatry, serving as a baseline for species-level divergence among allopatric populations [13]. However, divergence and speciation can also occur in the absence of obvious phenotypic divergence [12], especially in island systems where populations can evolve in isolation [9, 15]. Using genetic markers such as mtDNA, we can infer rates of gene flow, evolutionary isolation, and time since common ancestry [23, 24]. It may be tempting to use simple genotypic or phenotypic markers alone to determine species-level divergence, but completion of the speciation process may not be fully encapsulated (or diagnosed) this way [12, 24]. Here we do not delimit species, but rather use these two different divergence measures to explore patterns of divergence among largely co-distributed Philippine bird populations.

The Philippines are one of the world’s “hottest” biodiversity hotspots, hosting many endemic and threatened species and subspecies [25, 26]. Only 3% of original primary vegetation remains in the Philippines [25], where nearly half of all endemic species are threatened with extinction [26]. Many endemic subspecies are already extinct [27]. Thirty percent of bird species here are considered endemic, but nearly 80% of non-endemic species include multiple subspecies that are endemic to different islands [28]. Compared with mammals, amphibians, and freshwater fish, Philippine birds have significantly lower levels of species-level endemism [25]. However, evidence from a genetic study of 7 bird species suggested this endemism might be greatly underestimated [10]. Determining which endemic populations deserve full species status and which do not remains an unresolved issue [3, 10, 27, 29, 30], but our developing knowledge highlights the need for research with applications in both conservation and evolutionary biology. Here we examine genetic and phenotypic divergences within and among 48 species of Philippine birds, considering the patterns of these divergences and the cases in which lineages may have become full species.

## Materials and Methods

### Study Region and Sampling Design

In the Philippines, over 570 species of migrant and resident birds are distributed across a tropical archipelago stretching latitudinally along the Western Pacific Rim [28]. Over 7,000 islands make up the Philippines, and many islands host their own endemic species and subspecies [27]. Avian colonization and vicariance events in the Philippines are likely to have occurred multiple times and from multiple sources [31–33], and heavily restricted gene flow is common between at least some island populations [32, 34]. Unlike many mainland taxa, species ranges in the Philippines are likely to have been quite stable over time, having experienced little Pleistocene fluctuation [32].

During prior research, fieldwork archived museum specimens representing avian assemblages on 9 islands, representing 4 Pleistocene Aggregate Island Complexes, or PAICs [33], to provide a substantial degree of geographic co-distribution to our broad comparisons. This sampling scheme affected sample sizes within populations. Using museum specimens, we obtained between 1 and 11 individuals per population (average *n* = 4) from 117 populations, including 109 described subspecies of 48 Philippine bird species representing 31 families and 12 orders (Table A in S1 File). Our taxonomic sampling includes lineages with undifferentiated populations, lineages with recognized subspecific differentiation, and lineages with now-recognized full biological species. Thus, among lineages, our samples represent differentiation that spans the divergence and speciation process. Most orders are represented by only one species, whereas the order Passeriformes is represented by 34 species and 79 subspecies. Nearly all (98%) of the subspecies sampled are endemic to the Philippines. Since our study began, several of these species have been split by some authorities [35–37], but we have retained these comparisons because they are complementary and useful in assessing among-lineage divergence patterns in Philippine birds.

### Phenotypic Comparisons

We performed 96 pairwise phenotypic comparisons following the Tobias et al. [13] quantitative method for scoring plumage and morphometric characters. We are not using this method to delimit species. Rather, we are using it as a heuristic standard by which to gauge population divergence in phenotypic space; it provides a transparent and broadly applied methodology [36] that has some basic utility when comparing diverse lineages. Using this method, *minor*, *moderate*, *major*, and *exceptional* differences in plumage received scores of 1, 2, 3, and 4, respectively, and differences among morphometric characters were calculated as Cohen’s *d* effect sizes (the difference between means divided by standard deviation) for wing chord, tail length, tarsus length, and bill length between populations and scored as follows: |0.2–2| = *minor*, |2–5| = *moderate*, |5–10| = *major*, and |> 10| = *exceptional* [13]. Morphometric comparisons were performed among samples of *n* = 2–10 with equal sex ratios for all pairwise comparisons. Whenever possible only males were compared, and immature birds were not included in plumage or morphometric analyses. In total, 728 study skins were examined for phenotypic comparison (Table A in S1 File). We were unable to follow the recommendation from [13] to include vocal scores due to insufficient data being available, and we did not include scores for the presence or absence of hybrid zones, because most of the populations we studied are strictly allopatric. Because selection acts non-independently on morphometric characters more commonly than on plumage characters, each phenotypic pairwise comparison considers the 3 highest scores for plumage and only the single greatest increase and decrease in morphometric effect sizes between populations. Tobias et al. [13] recommended that total scores of 7 or more should be regarded as sufficiently divergent to be considered as full species. Despite the exclusion of vocal scoring and geographic structure, we also consider a phenotypic score of 7, conservatively, to be a conceptual threshold for identifying what we term phenotypically highly divergent taxa.

### Genotypic Comparisons

We performed 96 pairwise genetic comparisons using mtDNA sequence data from a single marker (ND2). DNA was extracted from fresh frozen tissues (or temporarily ethanol-preserved tissues for borrowed samples) from 491 individuals from 48 species of Philippine birds using Qiagen DNeasy blood and tissue extraction kits. The mitochondrial gene ND2 was amplified using standard PCR protocols with the forward primer L5215 [38] and reverse primer H6313 [39]. Sanger sequencing was performed by the University of Washington’s High Throughput Genomics lab (www.htseq.org) using the same primers and the following custom internal forward primers designed for this study; Eumy.ND2inter (ACAAAAACCCCAGCACTWAG), Hypsi.ND2inter (TAAACTCAATCAAAACCCTA), OtusND2inter (CCCAACCCTATTGACCMYAA), ParusND2inter (TTCTCCTCCATCTCCCACCT), Phapitre.ND2inter (CTACTAACCTTCTAYCTWTA), SittaND2inter (TATTAACCACCATAGCCATC), Zoster.ND2inter (CTACTCACATGCATAGCCGT), Collo.ND2inter (TCCCATCTCGGATGAATATC), Microhi.ND2inter (ATAATAATTACCTGAACAAA), and Phyllos.ND2inter (ACCGGRCTRCTMCTRTCCACA). Sequence data were visually inspected and cleaned when required then aligned using Sequencher 4.0. Partial ND2 sequences ranged in length from 692 to 1041 bp (average = 955 bp). Genetic divergence was calculated as Jukes-Cantor corrected *p*-distance in MEGA 5.0 [40], which derives the average distance between all individuals in the two sampled populations. In addition, MtDNA (ND2) haplotype networks were generated for 8 species with paraphyletic population relationships in DnaSP and Network 4.6.1.0 [41, 42]. GenBank accession numbers for all 491 individuals are provided in Table A in S1 File.

### Divergence Levels and Speciation Processes

We make a key conceptual distinction between divergence and speciation. Populations diverge before and after speciation; it is a continuous process. We therefore focus more on the process, though we consider divergence levels in both categorical and continuous ways (or semi-continuous in the case of phenotypic divergence scoring). We do not propose species limit thresholds in this study. Placing thresholds for species limits on genetic data is an inherently contentious issue, in part because no proposed divergence value clearly indicates the completion of speciation [20, 43, 44]. Here we have chosen a rather high threshold (5% corrected pairwise divergence in ND2) to be conservative in erecting a conceptual category that we term genetically highly divergent lineages, useful to highlight lineages for further studies on species limits. We have chosen this conservative threshold in part to include consideration of the variability of mutation rates among birds [45], especially at shallow levels of divergence [46–49]. Specifically, we set a genetic divergence threshold at 5% Jukes-Cantor corrected ND2 *p*-distance. The choice of 5% was done blindly with respect to the data, using a value that would be recognized as conservative among those generating the primary literature on species limits and genetic divergence. Using a generic mtDNA divergence rate of ~2% per million years [45, 50], this represents approximately 2.5 Myr of divergence. More than half of the 192 sister-species pairs considered by Weir and Schluter [51] had divergence levels below this threshold, and even lower threshold values have been proposed by others [52, 53]. We emphasize, however, that this is simply an indication of lineage divergence and not an indication of speciation. Genetic divergence on these terms occurs through neutral processes, whereas speciation is thought to be driven largely by selection [12, 54], and legitimate biological species can have lower genetic divergence values [51] while divergences of this threshold level can occur within a single biological species [55]. Indeed, there are numerous ways in which mtDNA can be misleading about species limits and relationships between populations [56–64].

Similarly, while we have used the methods of Tobias et al. [13] to provide a measure of phenotypic divergence, we do not apply their method to delimit species. We do, however, use their phenotypic divergence threshold of a phenotypic score of 7 or greater to denote what we call phenotypically highly divergent taxa. In terms of these genetic and phenotypic thresholds, population pairs were binned into four categories: *a)* those that are both phenotypically and genetically highly divergent (i.e., above these conceptual thresholds), *b)* those that are phenotypically highly divergent (i.e., a score of 7 or greater) but not genetically highly divergent, *c)* those that are highly genetically divergent (i.e., 5% genetic distance or greater) but not phenotypically highly divergent, and *d)* those populations whose divergence did not cross either conceptual high-divergence threshold. These bins represent different approaches to divergence and speciation (or lack thereof), and categorizing divergence among population pairs in such a way enabled us to test for heterogeneity among taxa. Specifically, we compared frequencies of Passeriformes versus non-Passeriformes species in each of the four bins, estimating *chi*-squared significance values assuming a null model of homogeneity. Separately from our categorical treatments, divergence data were examined as continuous (or semi-continuous) variables in divergence process space defined by phenotypic and genetic axes following [20].

With appropriate caution, we assume that populational divergences (phenotypic and genotypic) are independent between species, and we have a null expectation that these divergence processes will be independent with respect to higher taxonomy (and we emphasize that this assumption is *not* being applied to completion of the speciation process). In other words, we expect that divergence between populations within one species is not affected by similar processes occurring within another species, and we use a null model of phylogenetic independence for these processes (e.g., that divergence processes within species are not affected by what order the species is in). Note that this assumption or null model is independent of completed speciation; it considers divergence only. From reviews of genetic data we know this assumption to be violated in a strict sense (mutation rates vary among major avian lineages), but not to such a degree that it has prevented the utility and widespread application of this divergence metric [12]. For example, Weir and Schluter [45], in their review of another commonly used mtDNA gene cytochrome-*b*, found that “A molecular rate of approximately 2.1% (± 0.1%, 95% confidence interval) was maintained over a 12-million-year interval and across most of 12 taxonomic orders.” Similarly, we have chosen a phenotypic divergence metric that also has wide applicability [13], even though doubts remain about its universal effectiveness within the narrower scope of species delimitation [65, 66].

Without a reliable phylogeny that encompasses our sampled taxa, we cannot effectively test or correct for phylogenetic effects in our datasets, although we do examine such effects insofar as possible. Additionally, integrating genetic and phenotypic divergence data by making a bivariate plot in our divergence and speciation process space produced multiple pairwise comparisons within some species, introducing non-independence in some cases that confounds analyses. To correct for this when asking questions about possible higher-order effects, we averaged all within-species comparisons for species represented by more than two populations. We then used the corrected datasets to perform analyses of variance (ANOVA and MANOVA) to test for the effect of taxonomy on genetic and phenotypic divergence. Specifically, we tested for the effects of taxonomic order and family on divergence. Because many taxonomic orders were represented only by one species, we performed additional analyses treating all non-Passeriformes species as a single group (*n* = 14 species). Ordinary least squares regressions were also performed on single-lineage divergence estimates.

We do not have sufficient information to know whether all of our pairwise comparisons are between sister populations. To study the process of divergence and speciation within a lineage, it is common to focus on sister populations or taxa, and given our sampling it is likely that many inter-island comparisons are between sisters. However, when studying the divergence process among many lineages for overarching patterns of divergence, it is not necessary to include only sister lineages. In fact, sister populations are not needed to examine how genetic and phenotypic divergence generally proceed within a lineage. And, because sufficiently detailed intraspecific phylogenies do not yet exist for these 48 lineages (and this is not a phylogeographic study), our approach simply makes contrasts between and among populations. (We know that some comparisons are not between sisters, given that we have multiple populations of some species included, which we correct for when making among-lineage comparisons.) The presence of sister and non-sister lineages in the divergence process space does not materially affect the overarching understanding of how phenotype and genotype diverge—particularly among island populations such as these in which there is so little evidence of gene flow.

## Results

Genetic distances from 96 pairwise comparisons ranged from 0.02% to 12.5% (ND2 Jukes-Cantor corrected *p*-distance), and average divergence among populations was 3.16% (Table 1, Table B in S1 File). Phenotypic divergence scores from 96 comparisons ranged from 1 to 16 and averaged 6.0 (Table 1, Table B in S1 File). More than half of the species studied (25 of 48) revealed divergence levels surpassing our high-divergence thresholds either genetically, phenotypically, or both. In total, 42 pairwise comparisons (out of 96) were between populations that were highly divergent in either genotype or phenotype by our conceptual thresholds, suggesting the possibility of 29 species-level splits within 25 species (Tables B and C in S1 File). All 29 of these highly divergent populations are recognized as subspecies endemic to the Philippines [27]. In addition to comparisons of divergence, genetic analyses revealed mtDNA paraphly at the subspecific level in 8 species (Figure A in S2 File). ND2 haplotype relationships indicated undescribed cryptic populations within at least 4 taxa: *Accipiter virgatus confusus*, *Phapitreron leucotis brevirostris*, *Zosterops montanus vulcani*, and *Copsychus mindanensis mindanensis* (Figure A in S2 File). Comparisons within the remaining 40 species recovered haplotypically distinct populations, indicating a preponderance of genetic island endemism and lack of gene flow.

**Table 1:** Genetic distances (Jukes-Cantor corrected *p*–distance) and phenotypic scores (based on the Tobias et al. [13] method) for 96 pairwise comparisons. Results are binned into four categories: populations diverging across both genetic and phenotypic conceptual thresholds (A1-11), high phenotypic divergence (B1-23), high genetic divergence (C1-8), and populations with divergence levels that did not surpass either conceptual divergence threshold (D1-54). Case numbers correspond to Fig 1.

Case #	Population 1	Population 2	Genetic Distance	Phenotypic Score
**A1**	**Otus megalotis megalotis**	**O. m. everetti**	**5.20%**	**8**
**A2**	**Dicrurus hottenttotus palawanensis**	**D. h. samarensis**	**5.96%**	**7**
**A3**	**Rhipidura cyaniceps cyaniceps**	**R. c. albiventris**	**5.17%**	**7**
**A4**	**Orthotomus castaneiceps chloronotus**	**O. c. rabori**	**10.61%**	**10**
**A5**	**Irena cyanogastra cyanogastra**	**I. c. hoogstraali**	**6.22%**	**9**
**A6**	**Sitta oenochlamys apo**	**S. o. isarog**	**5.54%**	**8**
**A7**	**S. o. apo**	**S. o. mesoleuca**	**5.61%**	**9**
**A8**	**Eumyias panayensis panayensis**	**E. p. nigrimentalis**	**5.05%**	**8**
**A9**	**E. p. panayensis**	**E. p. nigriloris**	**5.63%**	**10**
**A10**	**E. p. nigrimentalis**	**E. p. nigriloris**	**5.15%**	**10**
**A11**	**Anthreptes malacensis birgitae**	**A. m. paraguae**	**9.46%**	**13**
**B1**	**Accipiter trivirgatus palawanus**	**A. t. extimus**	**3.04%**	**10**
**B2**	**Accipiter virgatus confusus (Luzon)**	**A. v. confusus (Panay)**	**0.66%**	**9**
**B3**	**A. v. confusus (Panay)**	**A. v. quagga**	**0.23%**	**8**
**B4**	**Phapitreron leucotis nigrorum**	**P. l. brevirostris (Mindanao)**	**3.55%**	**7**
**B5**	**P. l. brevirostris (Bohol)**	**P. l. nigrorum**	**3.07%**	**9**
**B6**	**P. l. leucotis**	**P. l. nigrorum**	**3.81%**	**8**
**B7**	**Dasylophus superciliosus superciliosus**	**D. s. cagayanensis**	**1.64%**	**8**
**B8**	**Otus megalotis megalotis**	**O. m. nigrorum**	**4.95%**	**10**
**B9**	**O. m. nigrorum**	**O. m. everetti**	**4.39%**	**11**
**B10**	**Halcyon coromanda linae**	**H. c. major**	**2.09%**	**11**
**B11**	**Aceros leucocephalus waldeni**	**A. l. leucocephalus**	**0.83%**	**11**
**B12**	**Chrysocolaptes lucidus haematribon**	**C. l. erythrocephalus**	**3.45%**	**15**
**B13**	**C. l. haematribon**	**C. l. montanus**	**2.52%**	**16**
**B14**	**C. l. erythrocephalus**	**C. l. montanus**	**2.72%**	**13**
**B15**	**Coracina striata striata**	**C. s. difficilis**	**1.65%**	**8**
**B16**	**Rhipidura superciliaris apo**	**R. s. samarensis**	**4.65%**	**7**
**B17**	**Phylloscopus trivirgatus nigrorum**	**P. t. mindanensis**	**4.87%**	**7**
**B18**	**P. t. benguetensis**	**P. t. mindanensis**	**4.63%**	**9**
**B19**	**Ficedula hyperythra dulangana**	**F. h. montigena**	**2.49%**	**9**
**B20**	**F. h. montigena**	**F. h. nigrorum**	**2.99%**	**9**
**B21**	**Diceaum trigonostigma cinereigulare**	**D. t. xanthopygium**	**4.39%**	**10**
**B22**	**Cinnyris jugularis obscurior**	**C. j. aurora**	**4.70%**	**9**
**B23**	**C. j. jugularis**	**C. j. aurora**	**4.54%**	**7**
**C1**	**Dicrurus hottentottus striatus**	**D. h. palawanensis**	**6.43%**	**6**
**C2**	**Corvus enca sierramadrensis**	**C. e. pusillus**	**7.18%**	**5**
**C3**	**Pycnonotus goiavier samarensis**	**P. g. suluensis**	**6.49%**	**4**
**C4**	**P. g. suluensis**	**P. g. goiavier**	**6.38%**	**3**
**C5**	**Ixos philippinus philippinus**	**I. p. guimarensis**	**12.49%**	**5**
**C6**	**I. p. saturatior**	**I. p. guimarensis**	**12.41%**	**5**
**C7**	**Sitta oenochlamys apo**	**S. o. oenochlamys**	**5.23%**	**6**
**C8**	**Prionochilus olivaceus parsonsi**	**P. o. olivaceus**	**5.07%**	**6**
**D1**	**Gallus gallus philippensis (Mind, Bohol, Cebu)**	**G. g. philippensis (Luzon)**	**0.02%**	**1**
**D2**	**G. g. philippensis (Mindanao, Bohol, Cebu)**	**G. g. philippensis (Busuanga)**	**0.02%**	**2**
**D3**	**Microhierax erythrogenys erythrogenys**	**M. e. maridonalis**	**4.19%**	**4**
**D4**	**Accipiter virgatus confusus (Luzon)**	**A. v. quagga**	**0.53%**	**6**
**D5**	**Phapitreron leucotis brevirostris (Mindanao)**	**P. l. brevirostris (Bohol)**	**1.64%**	**2**
**D6**	**P. l. brevirostris (Mindanao)**	**P. l. leucotis**	**3.01%**	**6**
**D7**	**P. l. brevirostris (Bohol)**	**P. l. leucotis**	**2.04%**	**6**
**D8**	**Ninox philippensis philippensis**	**N. p. centralis**	**3.03%**	**4**
**D9**	**Harpactes ardens ardens**	**H. a. herbeti**	**2.08%**	**5**
**D10**	**Buceros hydrocorax hydrocorax**	**B. h. mindanensis**	**2.25%**	**6**
**D11**	**Collocalia esculenta marginata**	**C. e. bagobo**	**2.81%**	**5**
**D12**	**Pitta erythrogaster erythrogaster**	**P. e. thompsoni**	**0.07%**	**3**
**D13**	**Pitta sordida sordida**	**P. s. palawanus**	**2.34%**	**5**
**D14**	**Gerygone sulphurea simplex**	**G. s. rhizophorae**	**0.30%**	**6**
**D15**	**Pachycephala philippinensis philippinensis**	**P. p. apoensis**	**3.78%**	**4**
**D16**	**Pachycephala albiventris albiventris**	**P. a. crissalis**	**0.63%**	**1**
**D17**	**Dicrurus balicassius balicassius**	**D. b. abraensis**	**1.34%**	**6**
**D18**	**Hypothymis azurea (Luzon)**	**H. azurea (Busuanga)**	**1.03%**	**4**
**D19**	**Parus elegans albescens**	**P. e. mindanensis**	**4.66%**	**4**
**D20**	**P. e. albescens**	**P. e. elegans**	**2.78%**	**2**
**D21**	**P. e. mindanensis**	**P. e. elegans**	**4.83%**	**4**
**D22**	**P. e. albescens**	**P. e. giliardi**	**2.44%**	**4**
**D23**	**P. e. mindanensis**	**P. e. giliardi**	**4.60%**	**5**
**D24**	**P. e. elegans**	**P. e. giliardi**	**0.53%**	**3**
**D25**	**P. e. albescens**	**P. e. montigenus**	**2.44%**	**3**
**D26**	**P. e. mindanensis**	**P. e. montigenus**	**4.57%**	**4**
**D27**	**P. e. elegans**	**P. e. montigenus**	**0.47%**	**2**
**D28**	**P. e. giliardi**	**P. e. montigenus**	**0.18%**	**2**
**D29**	**Pycnonotus urostictus urostictus**	**P. u. atricaudatus**	**4.45%**	**5**
**D30**	**P. u. urostictus**	**P. u. philippensis**	**3.19%**	**5**
**D31**	**P. u. atricaudatus**	**P. u. philippensis**	**4.67%**	**2**
**D32**	**P. u. urostictus**	**P. u. ilokensis**	**0.21%**	**4**
**D33**	**P. u. atricaudatus**	**P. u. ilokensis**	**4.23%**	**3**
**D34**	**P. u. philippensis**	**P. u. ilokensis**	**2.97%**	**6**
**D35**	**Pycnonotus goiavier samarensis**	**P. g. goiavier**	**0.43%**	**4**
**D36**	**Ixos philippinus philippinus**	**I. p. saturatior**	**0.98%**	**2**
**D37**	**Phylloscopus cebuensis cebuensis**	**P. c. luzonensis**	**2.28%**	**4**
**D38**	**Phylloscopus trivirgatus nigrorum**	**P. t. benguetensis**	**1.13%**	**5**
**D39**	**Zosterops montanus vulcani**	**Z. m. whiteheadi**	**2.12%**	**3**
**D40**	**Z. m. vulcani**	**Z. m. pectoralis**	**1.36%**	**5**
**D41**	**Z. m. whiteheadi**	**Z. m. pectoralis**	**2.09%**	**4**
**D42**	**Sitta oenochlamys oenochlamys**	**S. o. isarog**	**1.42%**	**4**
**D43**	**S. o. oenochlamys**	**S. o. mesoleuca**	**1.42%**	**6**
**D44**	**S. o. isarog**	**S. o. mesoleuca**	**0.21%**	**6**
**D45**	**Sarcops calvus melanonotus**	**S. c. calvus**	**0.32%**	**5**
**D46**	**Copyschus mindanensis mindanensis**	**C. m. deuteronymus**	**0.35%**	**1**
**D47**	**Ficedula hyperythra dulangana**	**F. h. nigrorum**	**1.33%**	**6**
**D48**	**Cyornis rufigastra philippinensis (Panay, Negros)**	**C. r. philippinensis (Mindanao)**	**0.57%**	**5**
**D49**	**C. r. philippinensis (Panay, Negros)**	**C. r. blythi**	**0.39%**	**2**
**D50**	**C. r. philippinensis (Mindanao)**	**C. r. blythi**	**0.51%**	**4**
**D51**	**Dicaeum hypoleucum pontifex**	**D. h. cagayanensis**	**3.28%**	**6**
**D52**	**Dicaeum australe (Luzon)**	**D. australe (Mindanao)**	**0.39%**	**6**
**D53**	**Cinnyris jugularis obscurior**	**C. j. jugularis**	**0.58%**	**2**
**D54**	**Lonchura leucogastra manueli**	**L. l. everetti**	**0.25%**	**3**

As expected given their recognized importance in generating Philippine biodiversity [33], genetic comparisons between PAICs were on average higher than those within PAICs. This relationship was significant both when testing 78 pairwise comparisons (*t* = 7.1, df = 72.2, *p* < 0.001; Table G in S1 File) and when testing a smaller subset of 10 species corrected for non-independence (i.e., each of these species’ among- or within-PAIC genetic distances are represented by a single value, the average of all respective comparisons for that taxon; *t* = 4.1, df = 9.8, *p* = 0.002; Table G in S1 File).

**Fig 1:**
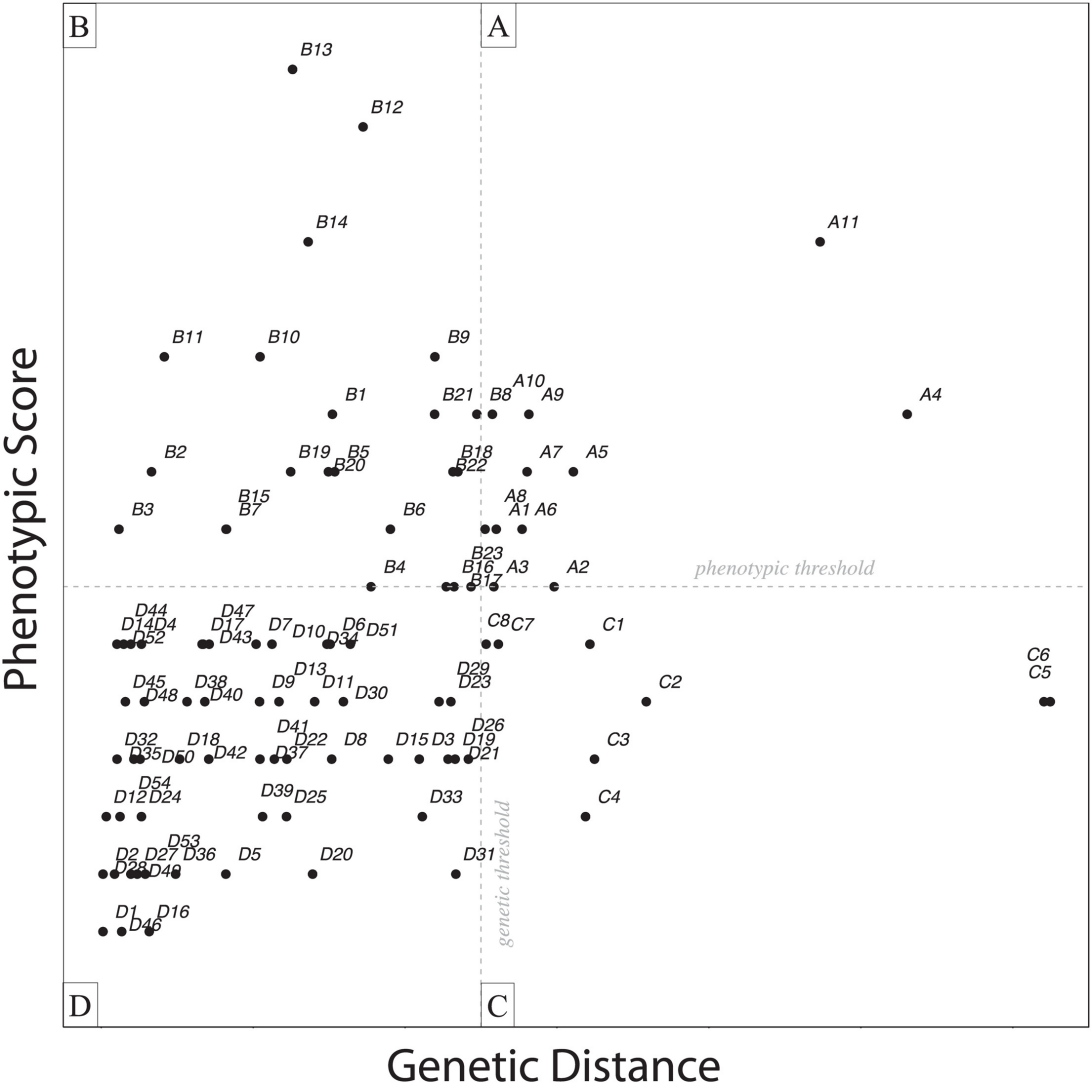
Phenotypic divergence, estimated as a quantitative score following Tobias et al. (2010), plotted against genetic divergence, estimated as Jukes-Cantor corrected *p*-distance, for 96 pairwise comparisons within 48 species. For binning purposes, thresholds of divergence are set at a phenotypic score of 7 and at 5% genetic divergence. These thresholds partition the results into 4 bins: A) Populations diverging across both genetic and phenotypic thresholds, B) high phenotypic divergence with lower genetic divergence, C) high genetic divergence with lower phenotypic divergence, and D) populations whose divergences did not surpass thresholds. Comparisons are labeled and the labels correspond to case numbers in Table 1.

**Fig 2:**
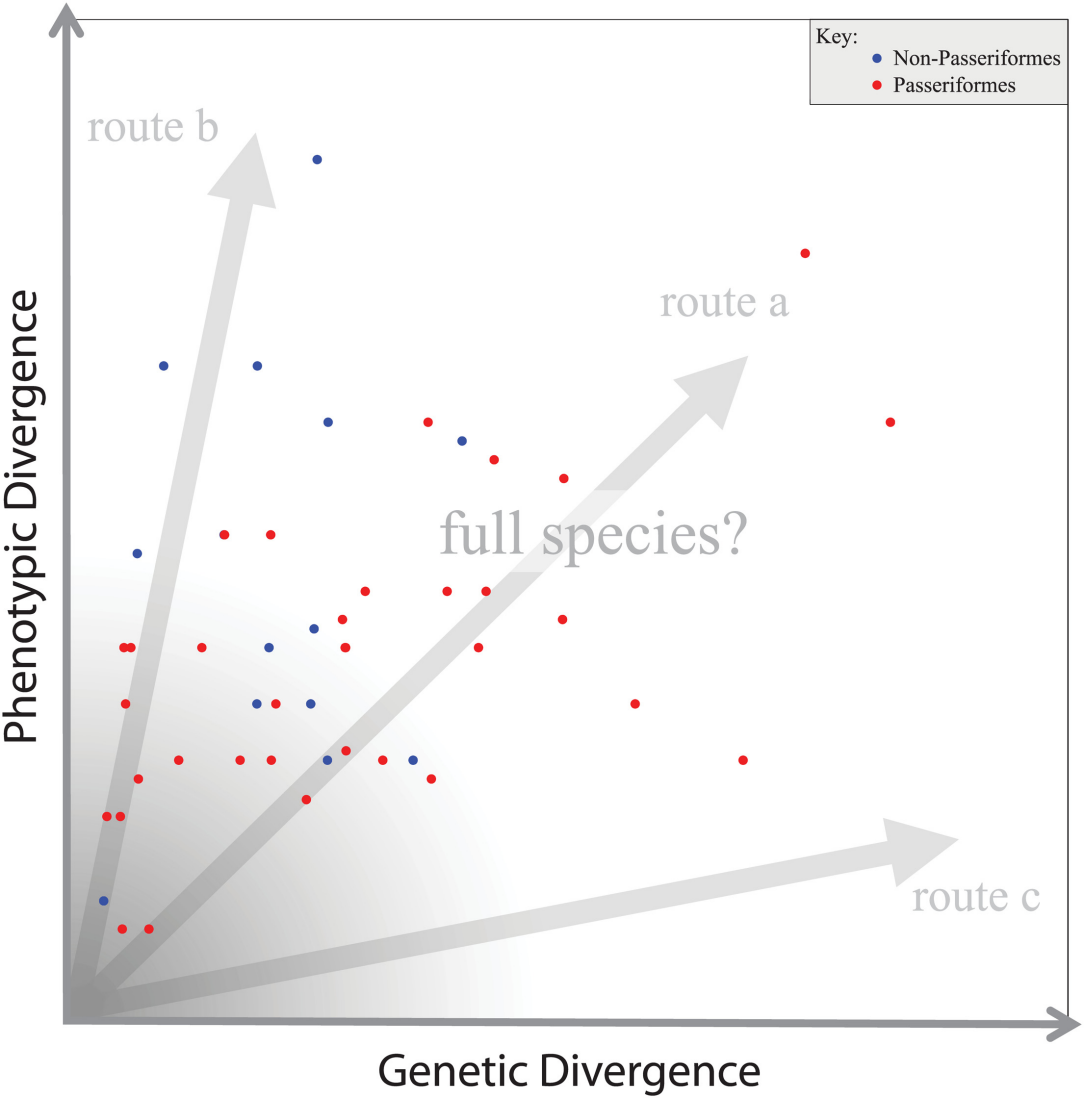
Speciation process space showing three possible general routes to speciation in relation to two axes: phenotypic divergence and genetic divergence. Units refer to phenotypic score (following the Tobias et al. (2010) method) and genetic *p*-distance (ND2), respectively. Pairwise comparisons within 48 species, corrected for non-independence within species, are plotted on this process space (blue = non-Passeriformes, red = Passeriformes). The large gray region near the origin indicates populations that have not surpassed conservative hypothetical speciation thresholds, whereas populations beyond the gray region are what may be full biological species not yet recognized as such. See Figure B in S2 File for specific identities for each point.

Integrating genotypic and phenotypic data in a bivariate divergence process space (Fig 1) enabled us to consider them together both in a continuous and discontinuous manner. For the latter, we erected bins representing our conceptual thresholds delimiting phenotypically and genetically highly divergent lineages and considered which pairwise comparisons crossed those thresholds. Many comparisons were highly divergent on both axes, crossing both genetic and phenotypic thresholds (Fig 1, bin A). These included 8 species (Table 1: A1-11), and this group represents one general divergence route toward speciation in this space (Fig 2, route a, even progression of divergence along both axes). There were 14 species (Table 1: B1-23) with populations that were highly divergent along the phenotypic axis alone (Fig 1, bin B), representing a second possible general route toward speciation (Fig 2, route b, rapid phenotypic divergence relative to genetic divergence), and 6 species (Table 1: C1-8) with populations that were highly divergent along the genetic axis alone (Fig 1, bin C), representing a third possible general divergence route toward speciation (Fig 2, route c, low phenotypic divergence relative to deeper genetic divergence). Of the 96 pairwise comparisons, 54 did not surpass either divergence threshold (though several approached these thresholds), and these were binned together in the lower regions of this divergence process space (Table 1: D1-54; Fig 1, bin D).

Relative frequencies of Passeriformes vs. non-Passeriformes populations were heterogeneous among bins. Only one non-Passeriformes species, *Otus megalotis*, occurred in bin A, and none occurred in bin C. However, in bin B non-Passeriformes outnumbered Passeriformes species, despite a lower frequency (27%) in the overall dataset. Phenotypically and genetically high-divergence (i.e., threshold-surpassing) levels occurred at similar frequencies among Passeriformes (19 and 18, respectively, out of 70 pairwise comparisons) but not among non-Passeriformes (15 and 1, respectively, out of 26 comparisons; Table 2). When comparing all 42 highly divergent pairwise comparisons together, frequencies of non-Passeriformes and Passeriformes were not significantly different than null expectations of homogeneity (i.e., taxonomy did not affect the overall number of comparisons considered to be highly divergent). However, when comparing either phenotypically or genetically highly divergent comparisons separately, frequencies of non-Passeriformes and Passeriformes were significantly different than null expectations (*P* < 0.05; Table 2, Table D in S1 File). In other words, more non-Passeriformes were phenotypically highly divergent than expected by chance, while more Passeriformes were genetically highly divergent than expected based on their frequencies in the overall dataset. Within individual bins, where sample sizes were small, frequencies of Passeriformes vs. non-Passeriformes were not significantly different than null expectations except for bin B, where the high occurrence of non-Passeriformes taxa was significant (*P* < 0.001; Table 2, Table D in S1 File). We note, however, that these tests were non-significant when the six species-level splits recognized by [35–37] were removed.

**Table 2:** Results of chi-squared tests of taxonomic heterogeneity among genetically and phenotypically highly divergent populations. For these tests all taxa were treated as either non-Passeriformes or Passeriformes. Expected frequencies were obtained from the frequencies of non-Passeriformes and Passeriformes comparisons in the overall dataset (27% and 73%, respectively; see Table F in S1 File). Bins A-D correspond to Fig 1.

Chi-squared test summaries					
Divergence Category	Non-Passeriformes	Passeriformes	df	Chi-sq	Significance
	(observed frequency)				
Highly divergent (Overall)	15	27	1	1.584	0.208
Highly divergent (Phenotypic)	15	19	1	5.013	0.025*
Highly divergent (Genetic)	1	18	1	4.581	0.032*
Bin A	1	10	1	1.803	0.179
Bin B	14	9	1	13.295	< 0.001 **
Bin C	0	8	1	2.971	0.085
Bin D (Not highly divergent)	11	43	1	1.1232	0.267

*significant at 95% confidence interval.

**significant at 99% confidence interval.

Pairwise comparisons among species, corrected for within-species non-independence (Table E in S1 File), were distributed across the 3 general routes of the speciation process space (Fig 2), with the majority of species appearing to follow the general divergence routes of a and b, with little representation along the general route c (i.e., few with low phenotypic divergence relative to deeper genetic divergence). Analyses of variance (ANOVA and MANOVA) on these data indicated significant effects from taxonomy (Passeriformes vs. non-Passeriformes) on divergence (Table 3). Taxonomic order and family, treated individually, did not significantly affect either phenotypic or genetic divergence (Table 3). When treating all non-Passeriformes orders as a single group, however, taxonomy significantly affected overall divergence (MANOVA, *P* < 0.01) and phenotypic divergence alone (ANOVA, *P* < 0.05), but not genetic divergence (ANOVA, *P* = 0.17; Table 3). As above, these effects were non-significant when the six species-level splits recognized by [35–37] were removed.

**Table 3:** Results of ANOVA and MANOVA, testing the effects of taxonomy on genetic and phenotypic divergence.

**ANOVA summaries**					
Effect of taxonomy on phenotypic divergence					
	df	Sum Sq	Mean Sq	F value	Significance
Non-Passeriformes / Passeriformes	1	27.9	27.9	4.62	0.047*
Taxonomic Order	10	132.3	13.23	2.19	0.08
Taxonomic Family	20	157.5	7.88	1.3	0.3
Residuals	16	96.6	6.04		
Effect of taxonomy on genetic divergence					
	df	Sum Sq	Mean Sq	F value	Significance
Non-Passeriformes / Passeriformes	1	10.9	10.92	2.04	0.17
Taxonomic Order	10	17.3	1.73	0.32	0.96
Taxonomic Family	20	173.6	8.68	1.62	0.17
Residuals	16	85.7	5.35		
**MANOVA Summary**					
Effect of taxonomy on overall divergence					
	df	Wilks' λ	Pillai's Trace^a^	Approx. F	Significance
Non-Passeriformes / Passeriformes	1	0.503	0.496	7.4	0.0058**
Taxonomic Order	10	0.266	-	1.41	0.19
Taxonomic Family	20	0.122	-	1.4	0.17
Residuals	16				

*significant at 95% confidence interval.

**significant at 99% confidence interval.

^a^ Pillai’s approximation given here, but *F-* and *P*-values are given for the Wilk’s approximation only.

Because these results suggesting effects at higher taxonomic levels might be affected by taxonomic philosophy and species limits concepts, we approached it in two other ways, by contrasting two taxonomic philosophies and by re-analyzing the data from a phylogenetic species concept perspective, in which we consider each reciprocally monophyletic mtDNA population to be a phylogenetic species, such that genetic independence equals an independent lineage against which to measure divergence (Table H in S1 File). The philosophical effect is arguably small, but the quantitative comparisons of phylogenetic species suggest the effect is not only real, but more extensive than the comparisons above suggest (Table H in S1 File, Table 4). Comparing only reciprocally monophyletic populations, and excluding the six species-level splits noted above, Passeriformes versus non-Passeriformes significantly affected genetic divergence (ANOVA, *P* < 0.024), and taxonomic order and family, treated individually, significantly affected phenotypic divergence (ANOVA, *P* < 0.04; Table 4). Overall divergence was affected by all three levels of higher taxonomy (MANOVA, *P* < 0.014; Table 4).

**Table 4:** Results of ANOVA and MANOVA, testing the effects of taxonomy on genetic and phenotypic divergence using only reciprocally monophyletic populations (and excluding lineages already considered full species) for comparisons (details in Table H in S1 File).

**ANOVA summaries**					
Effect of taxonomy on phenotypic divergence					
	df	Sum Sq	Mean Sq	F value	Significance
Non-Passeriformes / Passeriformes	1	3.84	3.80	0.93	0.34
Taxonomic Order	7	69.73	9.96	2.44	0.037*
Taxonomic Family	16	201.86	12.62	3.09	0.002**
Residuals	36	146.96	4.08		
Effect of taxonomy on genetic divergence					
	df	Sum Sq	Mean Sq	F value	Significance
Non-Passeriformes / Passeriformes	1	21.29	21.29	5.59	0.023*
Taxonomic Order	7	7.52	1.07	0.28	0.96
Taxonomic Family	16	69.48	4.34	1.14	0.36
Residuals	36	85.7	5.35		
**MANOVA Summary**					
Effect of taxonomy on overall divergence					
	df	Wilks' λ	Pillai's Trace^a^	Approx. F	Significance
Non-Passeriformes / Passeriformes	1	0.692	0.308	7.80	0.0016**
Taxonomic Order	7	0.476	0.541	2.25	0.014*
Taxonomic Family	16	0.220	1.004	2.42	0.0008***
Residuals	36				

*significant at 95% confidence interval.

**significant at 99% confidence interval.

***significant at 99.9% confidence interval.

^a^ Pillai’s approximation given here, but *F-* and *P*-values are given for the Wilk’s approximation only.

**Fig 3:**
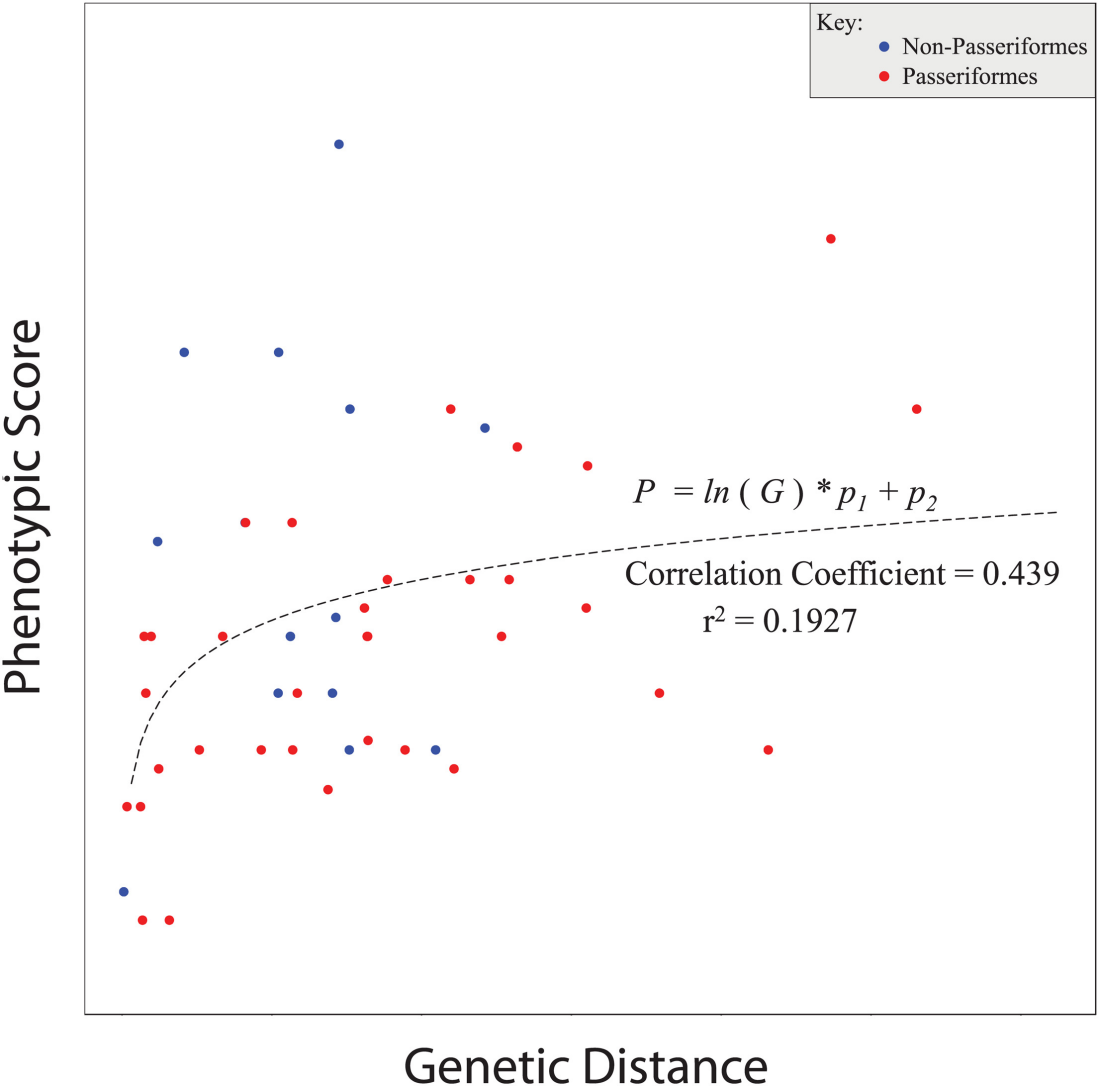
Nonlinear Least Squares Regression (see Table D in S1 File) and fitted curve showing apparent logarithmic increase of phenotypic divergence with respect to genetic divergence. In the equation *P = ln* (*G*) * *p*_*1*_ *+ p*_*2*_, “*P*” refers to phenotypic score and “*G*” refers to genetic distance.

Ordinary (linear) least squares regressions within each group (non-Passeriformes and Passeriformes) indicated that the slope of the relationship between genetic and phenotypic divergence was significantly different from zero for Passeriformes (*P <* 0. 001), but not for non-Passeriformes (*P* = 0.64; Table D in S1 File). Overall, phenotypic divergence was correlated with genetic divergence (linear regression *P* < 0.01), and it appeared to increase logarithmically (fitted nonlinear least squares regression *R*^2^ = 0.19; Fig 3, Table D in S1 File).

## Discussion

We found high levels of both phenotypic and genetic divergence and much cryptic diversity within the 48 species we studied, providing insights into the divergence and speciation process among these lineages and important ramifications for taxonomy and conservation.

### Divergence patterns

In considering how populations have diverged in the multidimensional speciation process space, two central results emerged. First, there may be higher-order taxonomic effects in how lineages are undergoing divergence; and, second, phenotypic divergence among lineages appears to have a logarithmic relationship with genetic divergence.

Support for heterogeneity among different lineages in the divergence or speciation processes occurring among them suggest that Passeriformes exhibit less phenotypic divergence relative to genetic divergence than other orders. Or, more precisely, the non-Passeriformes taxa in our study exhibited more plumage and morphometric divergence (traits likely under selection) relative to mitochondrial genetic divergence (likely to be neutral or nearly neutral) than Passeriformes taxa. These findings suggest that higher-order taxonomy probably affects divergence and speciation processes in this assemblage of lineages. For many birds, especially oscine Passeriformes, song can be more important for mate selection than plumage [67]. Because we did not measure vocalizations, phenotypic divergence in Passeriformes is probably underestimated here in the context of Tobias et al.’s [13] divergence quantification method. However, while large phenotypic divergences appeared in both Passeriformes and non-Passeriformes in this study, nearly all large genetic divergences (> 5%) appeared only in Passeriformes (Figs 1 and 2). Rates of mtDNA evolution are known to vary across taxa [45, 50, 68] and may be faster in Passeriformes than in other birds [46–49]. The heterogeneity in divergence patterns we observed between Passeriformes and non-Passeriformes might thus occur because of differential mtDNA mutation rates among birds and/or through underestimates of phenotypic divergence among oscine Passeriformes due to our exclusion of song. We also note that a different subset of Philippine birds (or a complete sampling), or using different high-divergence thresholds, might also affect these findings.

As noted in results, heterogeneity in higher-order taxonomic effects vanished when currently recognized full species were removed from analyses (18 lineages treated here as subspecies from 8 species, four each of Passeriformes and non-Passeriformes; Table B in S1 File). However, when we adopted a phylogenetic species approach higher-order taxonomic effects remained strong. Insofar as these questions involve lineage divergence in genetics and phenotype, examinations need not be restricted to divergences below the species level (particularly in a system where there is so little evidence of gene flow). Determining how and to what extent higher-order taxonomic effects affect the divergence process among Philippine birds will require more comprehensive study. This would ideally include more lineages both above and below the species level and more extensive genotypic and phenotypic data.

Overall, our data show an apparent logarithmic increase of phenotypic divergence with respect to genetic divergence (Fig 3, Table D in S1 File). This is of interest for two reasons. First, it suggests that speciation in this system might be dominated by selection promoting divergence rather than by a gradual accumulation of differences in allopatry (neutral processes would more likely be linear). Secondly, it suggests limits to phenotypic divergence over time in this assemblage of lineages. This is not unexpected, as there are likely to be evolutionary constraints on phenotypic divergence that exceed any constraints on the continued accumulation over time of putatively neutral genetic divergence. In other words, on the genetic axis there is relentless ongoing mutation promoting divergence, while on the phenotypic axis such predictably directional forces are lacking. Interestingly, Delmore et al. [69] found a similar relationship among an assemblage of North American migratory bird species. Such findings require further research into how phenotypic divergence accrues within and among lineages.

In considering the generalized routes of divergence toward speciation in Figs 1 and 2, we found substantial numbers of lineages that appear to have followed the general routes a and b. Population pairs that were both genetically and phenotypically highly divergent (Fig 1 bin A, Table 1 A1-12) are the most likely candidates for elevation to full biological species. Large genetic distances suggest long periods of evolutionary isolation, and large phenotypic differences imply divergent selection, although selection is difficult to disambiguate from phenotypic plasticity [70]. This group includes taxa with large divergences in the *Dicrurus hottentottus* and *Otus megalotis* complexes, species complexes that include multiple contentious subspecies and putative species [21, 35, 36, 71, 72]. Population pairs among 14 species that were highly divergent phenotypically but not genetically (Fig 1 bin B, Table 1 B1-19) also seem likely to have undergone divergent selection (with the caveat that phenotypic plasticity may influence these results). Although the species in this group did not exceed our genetic threshold of 5%, relatively large distances (e.g., 3–4%) separated many populations (e.g., Table 1 B16-23), and many may well be full biological species [13]. Some of the highly divergent populations in this group (e.g., within the *Phapitreron leucotis* and *Chrysocolaptes lucidus* species complexes) have recently been elevated to full species [36].

The third general route to speciation (c in Fig 2) was less well represented among our 48 lineages. Population pairs that were not phenotypically divergent but were separated by greater than 5% genetic distances (Fig 1, bin C) included 6 species of Passeriformes (Table 1, C1-8). The divergences observed here likely result from extended time in evolutionary isolation, as changes in mtDNA sequence data are usually interpreted to be neutral or nearly neutral [73, 74, 75]. Canalization of phenotypic characters, notably plumage color and pattern, and body size and shape, may contribute to the lack of phenotypic divergence observed between these oscine Passeriformes populations [20], as occurs in some other noteworthy avian genera (e.g., *Scytalopus*, *Empidonax*). The populations in this group (Table 1, C1-8) represent deep divergence between cryptic populations (e.g., *Ixos philippinus guimarasensis* separated from other subspecies by more than 12% genetic distance despite very similar phenotype) and may merit elevation to full biological species as recently done by Dickinson and Christidis [37].

### Initial taxonomic implications

While we are cautious about applying simplistic threshold values to determine species limits, our data suggest that cryptic species remain within many currently recognized species of Philippine birds. Using conservative conceptual speciation thresholds we found at least 29 populations, currently recognized as Philippine endemic subspecies, that may with further study warrant consideration as full biological species. Average genetic distance from 96 within-species comparisons was 3.15%, and distances less than this separate many sympatric species in well-studied mainland systems [20, 52]. Our results support the suggestion of Lohman et al. (2010) that the lower rates of endemism in birds (compared to other vertebrates) in the Philippines may be an artifact of misclassifying distinct island populations of birds as subspecies, rather than species. Lohman et al. [10] also predicted that, upon further investigation, a more accurate measure of endemism among birds in the Philippines may exceed 50%, as occurs in other terrestrial vertebrates [18]. With highly divergent populations occurring in more than half of the species we studied, our data provide empirical support for this prediction. Taxonomic revision, taking into consideration multiple types of comparison (e.g., genomic comparisons, phenotypic scoring, behavioral and ecological traits, etc.) is needed in this system. Uniting phenotypic and genetic datasets, as we have done here, will prove essential to such revisions.

### Implications for conservation

Taxonomic designation can have real-world consequences on the conservation of populations [19], and in the Philippines birds and other wildlife are severely threatened by anthropogenic forces [17, 18]. Our results reemphasize the urgent need for a reappraisal of Philippine avian diversity expressed by Peterson [29] and Lohman et al. [10]. Further research is also warranted on the 29 endemic populations in 25 species that our data suggest to be largely through the speciation process (e.g., estimates of gene flow and evaluation of traits promoting reproductive isolation). In addition, our results show surprisingly little haplotype sharing among populations (i.e., high rates of genetic endemism), revealing a level of diversity with important implications for management and conservation below the species level for populations that have likely not yet achieved speciation.

## Supporting Information

S1 File **Table A,** List of all specimens compared in this study including taxon identification, locality, field catalog numbers, museum voucher numbers, and GenBank accession numbers for ND2 sequences generated in this study. **Table B,** Genetic distances and phenotypic scores for all 96 pairwise comparisons in this study presented in taxonomic order. **Table C,** Potential splits in 25 species supported by highly divergent pairwise comparisons, presented in taxonomic order. **Table D,** Chi-squared tests for taxonomic heterogeneity among highly divergent lineages and among bins. **Table E,** Genetic and phenotypic divergence scores after correcting for non-independence. **Table F,** Genetic distances for all 96 pairwise comparisons in this study including sample size, ND2 fragment size, and standard deviation of genetic distance both uncorrected and Jukes-Cantor corrected *p*-distances. **Table G,** Genetic divergence within and among Pleistocene Aggregate Island Complexes (PAICs) for 10 species, corrected for non-independence. **Table H,** Genetic distances and phenotypic scores for all reciprocally monophyletic pairwise comparisons in this study presented in taxonomic order. **Note: Tables A-H are individual sheets in a single S1 File.**
(XLS)

S2 File **Figure A,** Haplotype networks showing mtDNA paraphyly between 14 subspecies of 8 species. **Figure B,** Plot of all 48 species included in the dataset with species labels. Data points here reflect data corrected for non-independence among species (see Table E in S1 File).
(PPT)

## References

[1] Mayr E (1963) *Animal Species and Evolution*. Harvard: Belknap Press of Harvard University.

[2] Hurlbert SH (1971) The nonconcept of species diversity: a critique and alternative parameters. Ecology 52: 577–586. 28973811 10.2307/1934145

[3] Dickinson EC, Bahr N, Dowsett R, Pearson D, Remsen V, Roselaar CS, et al (2004) *The Howard and Moore Complete Checklist of Birds of the World*. 3rd edition London: A & C Black.

[4] Wilson DE, Reeder DM, Eds. (2005) *Mammal species of the world*: *a taxonomic and geographic reference* (Vol. 2). Baltimore: Johns Hopkins University Press.

[5] Frost DR (2009) *Amphibian species of the World*: *an online reference*. Version 5.3 American Museum of Natural History, New York Available: http://research.amnh.org/vz/herpetology/amphibia/.

[6] Stevens GC (1989) The latitudinal gradient in geographical range: how so many species coexist in the tropics. Amer Natur 133: 240–256.

[7] Bradshaw CJ, Sodhi NS, Brook BW (2008) Tropical turmoil: a biodiversity tragedy in progress. Front Ecol Environ 7: 79–87.

[8] Joppa LN, Roberts DL, Myers N, Pimm SL (2011) Biodiversity hotspots house most undiscovered plant species. Proc Nat Acad Sci USA 108: 13171–13176. 10.1073/pnas.1109389108 21730155 PMC3156159

[9] Bickford D, Lohman D, Sodhi NS, Ng PKL, Meier R, Winker K, et al (2007) Cryptic species as a window on diversity and conservation. Trends Ecol Evol 22: 148–155. 17129636 10.1016/j.tree.2006.11.004

[10] Lohman DJ, Ingram KK, Prawiradilaga DM, Winker K, Sheldon FH, Moyle RG, et al (2010) Cryptic diversity in “widespread” southeast Asian bird species suggests that Philippine avian endemism is gravely underestimated. Biol Cons 143: 1885–1890.

[11] Sargis EJ, Campbell KK, Olson LE (2013) Taxonomic boundaries and craniometric variation in the treeshrews (Scandentia, Tupaiidae) from the Palawan faunal region. J Mamm Evol 21: 111–123.

[12] Price T (2008) *Speciation in Birds*. Englewood, Colorado: Roberts & Company.

[13] Tobias JA, Seddon N, Spottiswoode CN, Pilgrim JD, Fishpool LD, Collar NJ (2010) Quantitative criteria for species delimitation. Ibis 152: 724–746.

[14] Winker K, Haig SM, Eds. (2010) Avian subspecies Orn Monogr 67: i–viii, 1–200.

[15] MacArthur RH, Wilson EO (1967) *The Theory of Island Biogeography* (Vol. 1). Princeton: Princeton University Press.

[16] Steadman DW (2006). *Extinction and Biogeography of Tropical Pacific Birds*. University of Chicago Press.

[17] Donald P, Collar NJ, Marsden S, Pain D (2010) *Facing Extinction*: *the World's Rarest Birds and the Race to Save Them*. London: Poyser.

[18] Heaney LR, Regalado JC Jr (1998) *Vanishing Treasures of the Philippine Rainforest*. Chicago: The Field Museum.

[19] Ong PS, Afuang LE, Rosell-Ambal RG (2002) *Philippine biodiversity conservation priorities*: *A second iteration of the National Biodiversity Strategy and Action Plan*. Quezon City, Philippines: DENR-Protected Areas and Wildlife Bureau, CI-Philippines, UP CIDS and FPE.

[20] Winker K (2009) Reuniting genotype and phenotype in biodiversity research. BioScience 59: 657–665.

[21] Collar NJ (2011) Species limits in some Philippine birds including the Greater Flameback *Chrysocolaptes lucidus*. Forktail 27: 29–38.

[22] Rasmussen PC, Allen DNS, Collar NJ, Demeulemeester B, Hutchinson RO, Jakosalem PGC, et al (2012) Vocal divergence and new species in the Philippine Hawk Owl *Ninox philippensis* complex. Forktail 28: 1–20.

[23] Avise JC (1994) *Molecular Markers*: *Natural History and Evolution*. New York: Springer.

[24] Avise JC (2000) *Phylogeography*: *the History and Formation of Species*. Cambridge: Harvard University Press.

[25] Myers N, Mittermeier RA, Mittermeier CG, da Fonseca GAB, Kent J (2000) Biodiversity hotspots for conservation priorities. Nature 403: 853–858. 10706275 10.1038/35002501

[26] Conservation International (2008) Biological diversity in the Philippines (M. McGinley, Topic Editor) In *Encyclopedia of Earth* (Cleveland C. J., Editor). Washington, D.C.: Environmental Information Coalition, National Council for Science and the Environment www.eoearth.org/article/Biological_diversity_in_the_Philippines.

[27] Dickinson EC, Kennedy RS, Parkes KC (1991) *The Birds of the Philippines*: *an Annotated Checklist* (Vol. 12). Tring: British Ornithologists' Union.

[28] Kennedy R, Gonzalez P, Dickinson E, Miranda H Jr, Fisher T (2000) *A Guide to the Birds of the Philippines*. Oxford: Oxford University Press.

[29] Peterson AT (2006) Taxonomy is important in conservation: a preliminary reassessment of Philippine species-level bird taxonomy. Bird Cons Int 16: 155–173.

[30] Collar NJ (2007) Philippine bird taxonomy and conservation: a commentary on Peterson (2006). Bird Cons Intl 17: 103–113.

[31] Diamond JM, Gilpin ME (1983) Biogeographic umbilici and the origin of the Philippine avifauna. Oikos 41: 307–321.

[32] Jones A, Kennedy R (2008) Evolution in a tropical archipelago: comparative phylogeography of Philippine fauna and flora reveals complex patterns of colonization and diversification. Biol J Linn Soc 95: 620–639.

[33] Brown R, Siler CD, Oliveros CH, Esselstyn JA, Diesmos AC, Hosner PA, et al (2013) Evolutionary processes of diversification in a model island archipelago. Ann Rev Ecol Evol Syst 44:411–435.

[34] Oliveros CH, Moyle RG (2010) Origin and diversification of Philippine bulbuls. Mol Phylo Evol 54:822–83. 10.1016/j.ympev.2009.12.001 19995611

[35] Dickinson EC, Remsen JV Jr, Eds. (2013) *The Howard & Moore Complete Checklist of the Birds of the World*. 4^th^ edition, Vol. 1 Eastbourne: Aves Press.

[36] del Hoyo J, Collar NJ, Eds. (2014) *HBW and Birdlife International Illustrated Checklist of the Birds of the World*. Part 1 Barcelona: Lynx Edicions and Birdlife International.

[37] Dickinson EC, Christidis L, Eds. (2014) *The Howard & Moore Complete Checklist of the Birds of the World*. 4^th^ edition, Vol. 2 Eastbourne: Aves Press.

[38] Hackett SJ (1996) Molecular phylogenetics and biogeography of tanagers in the genus *Ramphocelus*. Mol Phylogen Evol 5: 368–382. 10.1006/mpev.1996.0032 8728395

[39] Johnson KP, Sorenson MD (1998) Comparing molecular evolution in two mitochondrial protein coding genes (cytochrome *b* and ND2) in the dabbling ducks (tribe: Anatini). Mol Phyl Evol 10:82–94. 10.1006/mpev.1997.0481 9751919

[40] Tamura K, Peterson D, Peterson N, Stecher G, Nei M, Kumar S (2011) MEGA5: Molecular Evolutionary Genetics Analysis using maximum likelihood, evolutionary distance, and maximum parsimony methods. Mol Biol Evol 28: 2731–2739. 10.1093/molbev/msr121 21546353 PMC3203626

[41] Bandelt HJ, Forster P, Röhl A (1999) Median-joining networks for inferring intraspecific phylogenies. Mol Biol Evol 16: 37–48. 10331250 10.1093/oxfordjournals.molbev.a026036

[42] Librado P, Rozas J (2009) DnaSP v5: A software for comprehensive analysis of DNA polymorphism data. Bioinformatics 25: 1451–1452. 10.1093/bioinformatics/btp187 19346325

[43] Moritz C, Cicero C (2004) DNA barcoding: promise and pitfalls. PLoS Biol 2: 1529–1529. 10.1371/journal.pbio.0020354 PMC519004 15486587

[44] Baker AJ, Tavares ES, Elbourne RF (2009) Countering criticisms of single mitochondrial DNA gene barcoding in birds. Mol Ecol Res 9: 257–268. 10.1111/j.1755-0998.2009.02650.x 21564985

[45] Weir JT, Schluter D (2008) Calibrating the avian molecular clock. Mol Ecol 17: 2321–2328. 10.1111/j.1365-294X.2008.03742.x 18422932

[46] Nabholz B, Glémin S, Galtier N (2009) The erratic mitochondrial clock: variations of mutation rate, not population size, affect mtDNA diversity across birds and mammals. BMC Evol Biol 9: 54 10.1186/1471-2148-9-54 19284537 PMC2660308

[47] Nabholz B, Künstner A, Wang R, Jarvis ED, Ellegren H (2011) Dynamic evolution of base composition: causes and consequences in avian phylogenomics. Mol Biol Evol 28: 2197–2210. 10.1093/molbev/msr047 21393604 PMC3144382

[48] Ho SY (2007) Calibrating molecular estimates of substitution rates and divergence times in birds. J Avian Biol 38: 409–414.

[49] Ho SY, Lanfear R, Bromham L, Phillips MJ, Soubrier J, Rodrigo AG, et al (2011) Time-dependent rates of molecular evolution. Mol Ecol 20: 3087–3101. 10.1111/j.1365-294X.2011.05178.x 21740474

[50] Lovette IJ (2004) Mitochondrial dating and mixed support for the “2% rule” in birds. Auk 121: 1–6.

[51] Weir JT, Schluter D (2007) The latitudinal gradient in recent speciation and extinction rates of birds and mammals. Science 315: 1574–1576. 17363673 10.1126/science.1135590

[52] Hebert PDN, Stoeckle MY, Zemlak TS, Francis CM (2004) Identification of birds through DNA barcodes. PLoS Biol 2: e31. 15455034 10.1371/journal.pbio.0020312 PMC518999

[53] Saitoh T, Sugita N, Someya S, Iwami Y, Kobayashi S, Kamigaichi H, et al (2015) DNA barcoding reveals 24 distinct lineages as cryptic bird species candidates in and around the Japanese Archipelago. Mol Ecol Res 15: 177–186. 10.1111/1755-0998.12282 24835119

[54] Coyne JA, Orr HA (2004) *Speciation*. Sunderland, USA: Sinauer Associates.

[55] Hogner S, Laskemoen T, Lifjeld JT, Porkert J, Albayrak T, Kabasakal B, et al **(**2012) Deep sympatric mitochondrial divergence without reproductive isolation in the common redstart *Phoenicurus phoenicurus*. Ecol Evol 2: 2974–2988. 10.1002/ece3.398 23301165 PMC3538993

[56] Irwin D J (2002) Phylogeographic breaks without geographic barriers to gene flow. Evolution 56: 2383–2394. 12583579 10.1111/j.0014-3820.2002.tb00164.x

[57] Cheviron ZA, Brumfield RT (2009) Migration-selection balance and local adaptation of mitochondrial haplotypes in rufous-collared sparrows (*Zonotrichia capensis*) along an elevational gradient. Evolution 63: 1593–1605. 10.1111/j.1558-5646.2009.00644.x 19187247

[58] Galtier N, Nabholz B, Glemin S, Hurst GDD (2009) Mitochondrial DNA as a marker of molecular diversity: a reappraisal. Mol Ecol 18: 4541–4550. 10.1111/j.1365-294X.2009.04380.x 19821901

[59] Ribeiro AM, Lloyd P, Bowie RCK (2011) A tight balance between natural selection and gene flow in a southern African arid-zone endemic bird. Evolution 65: 3499–3514. 10.1111/j.1558-5646.2011.01397.x 22133221

[60] Toews DP, Brelsford A (2012) The biogeography of mitochondrial and nuclear discordance in animals. Mol Ecol 21: 3907–3930. 10.1111/j.1365-294X.2012.05664.x 22738314

[61] Pavlova A, Amos JN, Joseph L, Loynes K, Austin J, Keogh JS, et al (2013) Perched at the mito-nuclear crossroads: divergent mitochondrial lineages correlate with environment in the face of ongoing nuclear gene flow in an Australian bird. Evolution 67: 3412–3428. 10.1111/evo.12107 24299397

[62] Peters J L, Winker K, Millam KC, Lavretsky P, Kulikova I, Wilson RE, et al (2014) Mito-nuclear discord in six congeneric lineages of Holarctic ducks (genus *Anas*). Mol Ecol 23: 2961–2974. 10.1111/mec.12799 24854419

[63] Dolman G, Joseph L (2015) Evolutionary history of birds across southern Australia: structure, history and taxonomic implications of mitochondrial DNA diversity in an ecologically diverse suite of species. Emu 115: 35–48.

[64] Morales HE, Pavlova A, Joseph L, Sunnucks P (2015) Positive and purifying selection in mitochondrial genomes of a bird with mitonuclear discordance. Mol Ecol 24: 2820–2837. 10.1111/mec.13203 25876460

[65] Winker K (2010) Is it a species? Ibis 152:679–682.

[66] Remsen JV Jr (2015) [Review of] HBW and BirdLife International Illustrated Checklist of the Birds of the World Volume 1: Non-passerines. J Field Orn 86:182–187.

[67] Gill FB (2007) *Ornithology*. 3^rd^ edition New York: W. H. Freeman and Company.

[68] Johnson KP, Seger J (2001) Elevated rates of nonsynonymous substitution in island birds. Mol Biol Evol 18: 874–881. 11319271 10.1093/oxfordjournals.molbev.a003869

[69] Delmore KE, Kenyon HL, Germain RR, and Irwin DE. 2015 Phenotypic divergence during speciation is inversely associated with differences in seasonal migration. Proc Roy Soc Lond B 282:20151921. 10.1098/rspb.2015.1921 PMC4685813 26559951

[70] Ghalambor CK, McKay JK, Carroll SP, Reznick DN (2007) Adaptive versus non-adaptive phenotypic plasticity and the potential for contemporary adaptation in new environments. Funct Ecol 21: 394–407.

[71] Allen D (2006) New records and other observations of birds on the island of Tablas, Romblon province, Philippines. Forktail 22: 77–84.

[72] Miranda HC Jr, Brooks DM, Kennedy RS (2011) Phylogeny and taxonomic review of Philippine lowland scops owls (Strigiformes): Parallel diversification of highland and lowland clades. Wilson J Orn 123:441–453.

[73] Avise JC, Ball RM, Arnold J (1988) Current versus historical population sizes in vertebrate species with high gene flow: a comparison based on mitochondrial DNA lineages and inbreeding theory for neutral mutations. Mol Biol Evol 5: 331–344. 3405076 10.1093/oxfordjournals.molbev.a040504

[74] Ball RM Jr, Avise JC (1992) Mitochondrial DNA phylogeographic differentiation among avian populations and the evolutionary significance of subspecies. Auk 109: 626–636.

[75] Galtier N, Nabholz B, Glémin S, Hurst GDD (2009) Mitochondrial DNA as a marker of molecular diversity: a reappraisal. Mol Ecol 18: 4541–4550. 10.1111/j.1365-294X.2009.04380.x 19821901

