# Supplementary material for: Integration of Genetic and Phenotypic Data in 48 Lineages of Philippine Birds Shows Heterogeneous Divergence Processes and Numerous Cryptic Species
Source: PLoS One. 2016 Jul 21;11(7):e0159325. doi: 10.1371/journal.pone.0159325 (PMC4956044; doi:10.1371/journal.pone.0159325)
Supplement: S2 File — Figure A, Haplotype networks showing mtDNA paraphyly between 14 subspecies of 8 species. Figure B, Plot of all 48 species included in the dataset with species labels. Data points here reflect data corrected for non-independence among species (see Table E in S1 File). (PPT) [file pone.0159325.s002.ppt]

## Slide 1
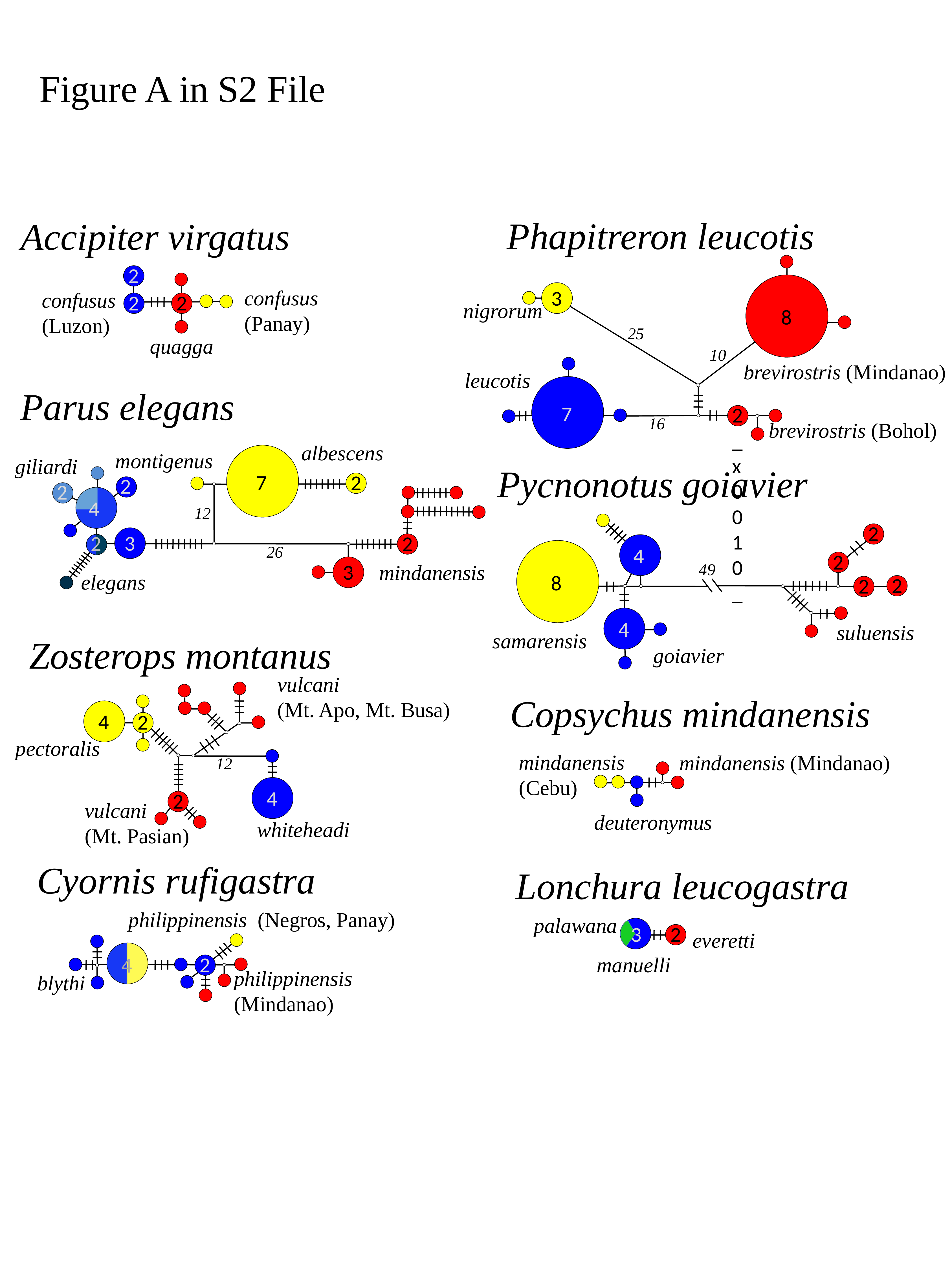

Figure A in S2 File
Phapitreron leucotis
3
8
25
10
7
2_x0010_
16
nigrorum
brevirostris (Mindanao)
leucotis
brevirostris (Bohol)
Accipiter virgatus
2
2
2
confusus
(Panay)
confusus
(Luzon)
quagga
Parus elegans
7
2
2
3
2
26
3
12
montigenus
giliardi
2
2
mindanensis
albescens
Pycnonotus goiavier
2
4
2
49
8
2
2
4
suluensis
samarensis
goiavier
4
elegans
Zosterops montanus
4
2
12
4
2
vulcani
(Mt. Apo, Mt. Busa)
pectoralis
vulcani
(Mt. Pasian)
whiteheadi
Copsychus mindanensis
mindanensis
(Cebu)
mindanensis (Mindanao)
deuteronymus
Cyornis rufigastra
Lonchura leucogastra
palawana
3
2
everetti
manuelli
philippinensis (Negros, Panay)
2
4
philippinensis
(Mindanao)
blythi

## Slide 2
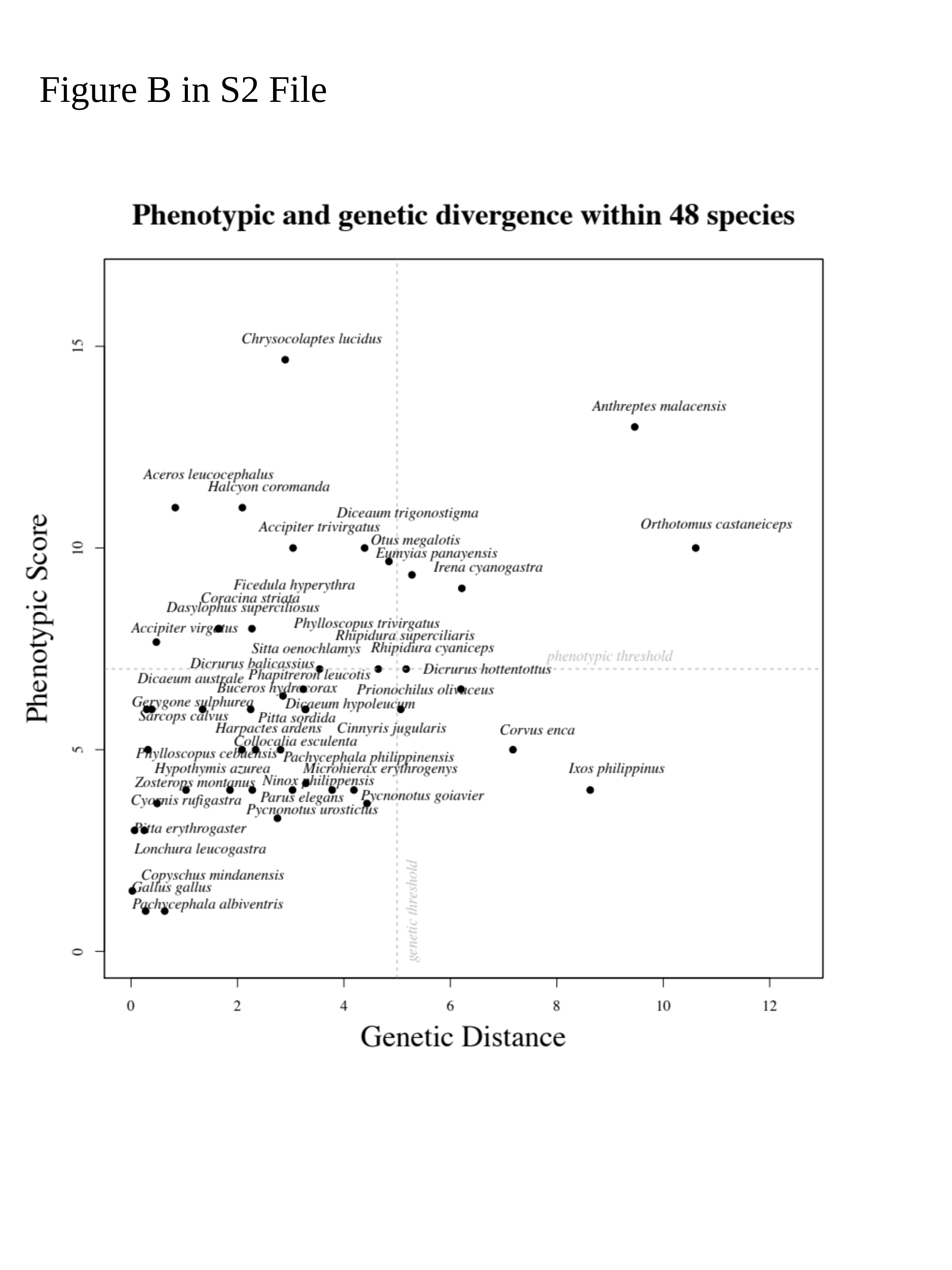

Figure B in S2 File
